# A cross-sectional analysis of coffee intake and hypertension prevalence: results from the NHANES 2005–2020

**DOI:** 10.3389/fnut.2025.1615528

**Published:** 2025-08-11

**Authors:** Hua Li, Wei Chen, Zao Zeng, Ning Ding, Ju Luo, Keng Li

**Affiliations:** ^1^Department of Critical Care Medicine, Shenzhen Bao'an District Songgang People's Hospital, Shenzhen, China; ^2^Department of Gerontology, The Affiliated Changsha Central Hospital, Hengyang Medical School, University of South China, Changsha, Hunan, China; ^3^Department of Emergency, The Affiliated Changsha Central Hospital, Hengyang Medical School, University of South China, Changsha, Hunan, China; ^4^Department of Emergency, The Seventh Affiliated Hospital, Hengyang Medical School, University of South China (Hunan Provincial Veterans Administration Hospital), Changsha, Hunan, China

**Keywords:** hypertension, risk, NHANES, cross-sectional study, coffee

## Abstract

**Objective:**

To investigate the association between coffee consumption and hypertension risk.

**Methods:**

Using data from the 2005–2020 National Health and Nutrition Examination Survey (NHANES) on 41,685 adults, multivariable logistic regression models examined the relationship between categorical coffee intake (none, >0 to < 1, ≥1 to < 2, ≥2 to < 3, ≥3 to < 4, and ≥4 cups/day) and hypertension, with stratified and curve-fitting analyses.

**Results:**

Compared to non-consumers, moderate daily intake of 1–3 cups was significantly associated with lower hypertension odds (OR 0.829–0.869, *p* < 0.05), more prominently in those < 60 years (OR 0.957, 95% CI 0.940–0.975). Curve fitting revealed a U-shaped association between coffee consumption and hypertension risk.

**Conclusion:**

While a moderate coffee intake (1–3 cups/day) was associated with a lower prevalence of hypertension, especially among adults under 60 years, this cross-sectional study cannot establish causality. Further prospective studies are needed to confirm these findings.

## Introduction

Hypertension is a significant factor in cardiovascular diseases, which are the most common cause of morbidity and mortality worldwide (1, 2). The global frequency of hypertension has progressively increased over the last few decades, with an estimated 1.3 billion persons afflicted in 2019 (3). Identifying modifiable risk factors and lifestyle interventions that can help prevent or manage hypertension is crucial for reducing the global burden of cardiovascular diseases (4, 5).

Coffee serves as one of the most extensively consumed beverages globally (6), according to estimates, the daily consumption of coffee is anticipated to exceed 2.25 billion cups (7). Due to its extensive use, there is significant interest in comprehending the possible influence of coffee on health consequences. Multiple research have examined the correlation between coffee intake and different health issues, such as type 2 diabetes (8), cardiovascular diseases (9), neurological disorders (10), and specific forms of cancer (11, 12).

Several meta-analyses have suggested that habitual coffee intake may be linked to a decreased likelihood of developing type 2 diabetes (13–15). Additionally, several studies have indicated that consuming coffee may reduce the likelihood of developing cardiovascular disorders, including (16) and stroke (17). Furthermore, coffee consumption has been known to be associated with a reduced likelihood of acquiring neurodegenerative conditions such as Alzheimer's disease and Parkinson's disease (18).

However, the relationship between coffee consumption and hypertension remains less clear. Several studies have indicated that consuming coffee may elevate blood pressure and heighten the likelihood of developing hypertension, potentially as a result of caffeine's immediate influence on vascular resistance and sympathetic nervous system activation (19). On the other hand, other studies have found no significant association or even a negative correlation between coffee consumption and the likelihood of developing hypertension (20, 21). Notably, a recent meta-analysis concluded that habitual coffee consumption was associated with statistically significant reduction in blood pressure in a dose-response manner (22). Similarly, other studies have observed moderate coffee intake was linked to the lowest hypertension risk (23, 24), Despite these findings, inconsistencies remain regarding the dose-response relationship, the influence of individual characteristics (e.g., age, sex, predisposition), and the role of different coffee components.

Coffee contains several bioactive compounds that may influence blood pressure regulation. In addition to caffeine, coffee is rich in polyphenols, particularly chlorogenic acid, which has been reported to exhibit antioxidant and anti-inflammatory properties and may improve endothelial function and reduce arterial stiffness (25, 26). Other micronutrients present in coffee, such as potassium and magnesium, have also been linked to blood pressure control (27, 28). The interactions among these constituents and their net effect on cardiovascular health are still being explored.

Given the inconsistent findings in the literature and the complex composition of coffee, further investigation is needed to clarify the relationship between coffee consumption and hypertension. The objective of our study is to enhance the current understanding of the correlation between coffee intake and the risk of hypertension and to provide insights into the possible role of coffee as a modifiable lifestyle feature for the prevention and management of hypertension. The results of this research could have significant implications for public health recommendations and future investigations into the impact of coffee intake on health, particularly in the context of cardiovascular health.

## Methods

### Study population

This cross-sectional analysis utilized data from eight successive cycles of the National Health and Nutrition Examination Survey (NHANES) conducted between 2005 to 2020. NHANES is a nationally representative survey of the non-institutionalized U.S. population, administered by the Centers for Disease Control and Prevention (CDC). It employs a complex, multistage probability sampling design that includes stratification, clustering, and oversampling of specific subpopulations to improve estimate precision. Participants are drawn from all 50 U.S. states through geographically diverse sampling units. To minimize the possibility of duplicate inclusion of participants across cycles, we treated NHANES as repeated cross-sectional datasets. Each cycle includes a new, independent sample, and unique participant identifiers (sequence numbers) are assigned for each cycle. Therefore, individuals are unlikely to be included more than once.

**Figure 1:**
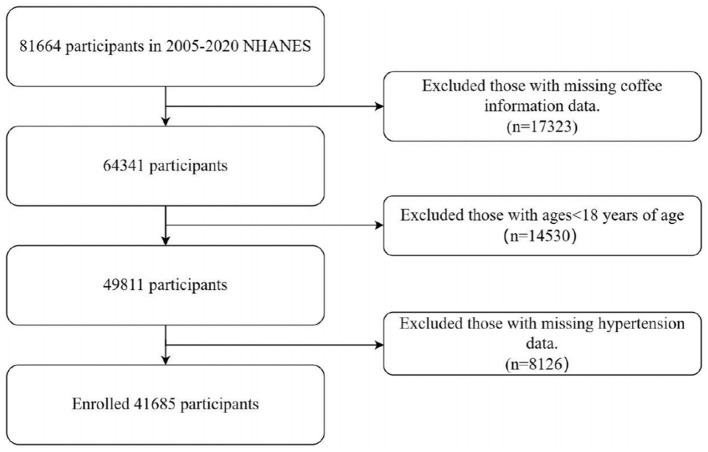
Flowchart in selecting the studying participants.

The study protocol obtained clearance from the ethics review board of the National Center for Health Statistics, and all subjects granted informed consent (29). Adherence to the Strengthening the Reporting of Observational Studies in Epidemiology (STROBE) principles was verified. NHANES dietary intake data were collected using two non-consecutive 24-h dietary recall interviews. The first recall was conducted in person at the Mobile Examination Center (MEC), while the second was completed via telephone 3 to 10 days later. NHANES sampling is designed to ensure that dietary recalls are distributed across all days of the week, including both weekdays and weekends, although the combination of recall days may vary among participants. Participants were excluded if they had incomplete coffee consumption data from the dietary recalls (*n* = 17,323), were under the age of 18 years (*n* = 14,530), or had missing hypertension information (*n* = 8,126). The final analytic sample included 41,685 participants (Figure 1).

### Coffee consumption data collection

Data on coffee consumption were derived from food frequency surveys and two 24 was identified using specific 8-digit food codes (starting with 921) from the Food and Nutrition Database for Dietary Studies (FNDDS) (30). Participants with two dietary recalls were selected, and the average amount of coffee consumed across recalls was calculated. A standard cup of coffee was defined as approximately 177 milliliters Consumption levels were categorized into six groups: no intake, >0 to < 1, ≥1 to < 2, ≥2 to < 3, ≥3 to < 4, and ≥4 cups/day.

### Definition of hypertension

Hypertension was characterized by the presence of one or more of the following criteria: current antihypertensive medication use, a prior physician diagnosis, four measurements of an average systolic blood pressure of at least 140 mmHg or a diastolic blood pressure of at least 90 mmHg.

### Covariates

Covariates were selected for their potential confounding effect on the coffee-hypertension relationship. Demographics included age, sex (male, female), race/ethnicity (Non-Hispanic White, Non-Hispanic Black, Mexican American, Other Hispanic, Other Race), education level (less than high school, high school or equivalent, college or above), and socioeconomic status (poverty-to-income ratio). Lifestyle-related covariates included dietary intake variables—total energy, calcium, phosphorus, magnesium, potassium, and sodium—which were extracted using the nhanesR R package (version 4.4.2) based on 24-h dietary recall data and the FNDDS. Additional covariates included physical activity (active, inactive, missing), alcohol consumption (no, yes, missing), smoking status (never, past, current, missing), sleep quality (no trouble, trouble, missing), and supplement intake (no, yes, missing). Health-related factors included body mass index (BMI), diabetes status (no, yes, missing), chronic kidney disease (CKD) (no, yes, missing) as per the kidney disease: improving global outcomes guidelines, and hyperlipidemia (no, yes, missing) based on cholesterol measurements.

### Statistical analysis

In this study, participants were categorized based on their hypertension status. The Kruskal-Wallis rank test was employed to analyze differences in baseline characteristics for continuous variables, whereas Fisher's exact test was used for categorical data. Coffee consumption was analyzed both categorically (no intake, >0 to < 1, ≥1 to < 2, ≥2 to < 3, ≥3 to < 4, and ≥4 cups/day) and continuously (cups/day). The correlation between coffee consumption and hypertension was investigated through logistic regression models: the Crude Model without adjustments, Model I adjusted for age, sex, race, and ethnicity, and Model II† and II‡ fully adjusted for all covariates. Stratified analysis and smooth curve fitting were applied to delve deeper into the coffee-hypertension association. To assess potential non-linear and threshold effects, a segmented regression model was used. The threshold level (i.e., turning point) was determined using a trial-and-error approach, in which candidate turning points were selected along a pre-defined interval of coffee consumption, and the point yielding the maximum model likelihood was chosen. To address missing data, two methods were employed: a complete case analysis using multivariable logistic regression (Model II†) for participants without missing data (*n* = 22,104), and multiple imputation (Model II‡) to mitigate bias from missing values (*n* = 41,685). The odds ratio (OR) was calculated along with a 95% confidence interval (CI), with no intake as the reference. Results are presented as OR coefficients with 95% CI, *p*-values ≤ 0.05 are regarded as statistically significant. Data analyses were performed using R software (version 4.2.0; R Project) and EmpowerStats (EmpowerStats.com; X&Y Solutions Inc.).

## Results

Table 1 displays notable disparities in the baseline characteristics between the hypertensive (*n* = 16,017) and non-hypertensive (*n* = 25,668) groups. Hypertensives had higher mean daily coffee intake (1.27 ± 2.14 vs. 0.96 ± 1.96 cups, *P* < 0.001), elevated BMI (31.2 ± 7.4 vs. 27.4 ± 6.9 kg/m^2^, *P* < 0.001), and older age (59.5 ± 15.2 vs. 38.3 ± 18.3 years, *P* < 0.001). Differences were also observed in sex, race/ethnicity, education, smoking, sleep quality, diabetes (32.1% vs. 8.0%), alcohol consumption, physical activity, kidney disease (30.1% vs. 9.2%), and hyperlipidemia (79.6% vs. 57.0%, all *P* < 0.001). A higher proportion of hypertensives reported ≥1 cup/day coffee intake (40.1% vs. 29.2%). Dietary supplement use was higher among hypertensives (59.0% vs. 45.1%, *P* < 0.001), with notable differences in energy and nutrient intakes, highlighting the need to adjust for potential confounders in coffee-hypertension analyses.

**Table 1:** Characteristics of the study population based on the presence of hypertension.

**Variable**	**Overall (*n =* 41,685)**	**With hypertension (*n =* 16,017)**	**No hypertension (*n =* 25,668)**	***P*-value**
Coffee consumption, cup/d	1.076 ± 2.033	1.270 ± 2.137	0.955 ± 1.956	< 0.001
BMI, (kg/m^2^)	28.832 ± 7.385	31.217 ± 7.448	27.357 ± 6.947	< 0.001
Energy, kcal/d	2,103.278 ± 989.441	1,993.037 ± 946.861	2,172.070 ± 1,009.040	< 0.001
Calcium, mg/d	923.687 ± 582.787	868.530 ± 548.542	958.106 ± 600.620	< 0.001
Phosphorus, mg/d	1,330.384 ± 673.452	1,266.197 ± 636.387	1,370.438 ± 692.585	< 0.001
Magnesium, mg/d	289.350 ± 148.019	280.846 ± 142.220	294.657 ± 151.285	< 0.001
Potassium, mg/d	2,563.514 ± 1,257.641	2,514.507 ± 1,215.129	2,594.094 ± 1,282.530	< 0.001
Sodium, mg/d	3,421.605 ± 1,810.640	3,268.330 ± 1,734.807	3,517.250 ± 1,850.001	< 0.001
Age, years	46.467 ± 20.057	59.533 ± 15.192	38.314 ± 18.340	< 0.001
PIR %	2.504 ± 1.626	2.504 ± 1.589	2.504 ± 1.648	0.998
Sex, %				0.002
Female	21,754 (52.187%)	8,203 (51.214%)	13,551 (52.793%)	
Male	19,931 (47.813%)	7,814 (48.786%)	12,117 (47.207%)	
**Race and ethnicity, %**				< 0.001
Non-Hispanic Black	9,655 (23.162%)	4,537 (28.326%)	5,118 (19.939%)	
Non-Hispanic White	17,167 (41.183%)	7,030 (43.891%)	1,0137 (39.493%)	
Other Hispanic	3,916 (9.394%)	1,339 (8.360%)	2,577 (10.040%)	
Mexican American	6,404 (15.363%)	1,777 (11.094%)	4,627 (18.026%)	
Other Race - Including Multi-Racial	4,543 (10.898%)	1,334 (8.329%)	3,209 (12.502%)	
**Education, %**				< 0.001
Less than high school	5,443 (13.057%)	1,662 (10.376%)	3,781 (14.730%)	
High school or equivalent	14,800 (35.504%)	6,322 (39.471%)	8,478 (33.029%)	
College or above	21,082 (50.575%)	7,999 (49.941%)	13,083 (50.970%)	
Missing	360 (0.864%)	34 (0.212%)	326 (1.270%)	
**Smoking, %**				< 0.001
Never	21,843 (52.400%)	8,029 (50.128%)	13,814 (53.818%)	
Past	9,333 (22.389%)	4,986 (31.129%)	4,347 (16.935%)	
Current	7,335 (17.596%)	2,911 (18.174%)	4,424 (17.235%)	
Missing	3,174 (7.614%)	91 (0.568%)	3,083 (12.011%)	
**Trouble sleeping, %**				< 0.001
No	29,338 (70.380%)	10,416 (65.031%)	18,922 (73.718%)	
Yes	10,353 (24.836%)	5,592 (34.913%)	4,761 (18.548%)	
Missing	1,994 (4.783%)	9 (0.056%)	1,985 (7.733%)	
**Diabetes, %**				< 0.001
No	33,830 (81.156%)	10,827 (67.597%)	23,003 (89.617%)	
Yes	7,185 (17.236%)	5,134 (32.053%)	2,051 (7.990%)	
Missing	670 (1.607%)	56 (0.350%)	614 (2.392%)	
**Alcohol drinking, %**				< 0.001
Never	5,042 (12.095%)	2,076 (12.961%)	2,966 (11.555%)	
Current	24,894 (59.719%)	9,349 (58.369%)	15,545 (60.562%)	
Past	4,850 (11.635%)	2,706 (16.895%)	2,144 (8.353%)	
Missing	6,899 (16.550%)	1,886 (11.775%)	5,013 (19.530%)	
**Activity, %**				< 0.001
Inactive	6,726 (16.135%)	2,794 (17.444%)	3,932 (15.319%)	
Active	22,837 (54.785%)	7,944 (49.597%)	14,893 (58.022%)	
Missing	12,122 (29.080%)	5,279 (32.959%)	6,843 (26.660%)	
**CKD**				< 0.001
No	30,658 (73.547%)	10,362 (64.694%)	20,296 (79.071%)	
Yes	7,186 (17.239%)	4,828 (30.143%)	2,358 (9.187%)	
Missing	3,841 (9.214%)	827 (5.163%)	3,014 (11.742%)	
**Hyperlipidemia**				< 0.001
No	14,299 (34.303%)	3,262 (20.366%)	11,037 (42.999%)	
Yes	27,382 (65.688%)	12,752 (79.615%)	14,630 (56.997%)	
Missing	4 (0.010%)	3 (0.019%)	1 (0.004%)	
**Coffee consumption categorical, cup/d**				< 0.001
No intake	23,866 (57.253%)	7,988 (49.872%)	15,878 (61.859%)	
>0, < 1	3,391 (8.135%)	1,383 (8.635%)	2,008 (7.823%)	
≥1, < 2	5,740 (13.770%)	2,645 (16.514%)	3,095 (12.508%)	
≥2, < 3	3,786 (9.082%)	1,765 (11.020%)	2,021 (7.847%)	
≥3, < 4	2,010 (4.822%)	919 (5.738%)	1,091 (4.250%)	
≥4	2,892 (6.938%)	1,317 (8.223%)	1,575 (6.136%)	
**Supplement taken**				< 0.001
No	20,644 (49.542%)	6,556 (40.952%)	14,088 (54.900%)	
Yes	21,026 (50.458%)	9,453 (59.048%)	11,573 (45.100%)	

Data are presented as mean ± SD for continuous variables or *n* (%) for categorical variables, *P*-value: Kruskal Wallis Rank Test for continuous variables, Fisher Exact for categorical variables. BMI, body mass index; CKD, chronic kidney disease. PIR, poverty to income ratio.

Multiple logistic regression models (Table 2) were utilized to investigate the relationship between coffee intake and the risk of hypertension. The crude model revealed a notable inverse connection (OR 0.925, 95% CI 0.916–0.935 per cup/day increment). However, after adjusting for confounders in Models I and II†, this association was attenuated to non-significance (Model I: OR 0.996, 95% CI 0.985–1.007; Model II†: OR 0.999, 95% CI 0.983–1.014 per cup/day increment). Categorized analysis unveiled a potential non-linear relationship, with individuals consuming >0 to < 1 cup/day (OR 0.851, 95% CI 0.772–0.939) and ≥1 to < 2 cups/day (OR 0.829, 95% CI 0.770–0.892) exhibiting significantly reduced odds of hypertension compared to non-consumers in the fully adjusted Model II†. Higher intake levels (≥2 cups/day) were not linked to a statistically significant risk reduction. In Model II‡, accounting for missing data through multiple imputation, coffee consumption demonstrated a borderline significant protective effect (OR: 0.988, 95% CI: 0.976–1.000, *P* = 0.047583). The categorized analysis revealed significant protective effects for intake levels up to 3 cups/day compared to no intake (OR ranging from 0.829 to 0.857, *P* < 0.05), with the protective effect diminishing at higher intake levels (≥3 cups/day, OR closer to 1, *P* > 0.05).

**Table 2:** Relationship between coffee and hypertension in different models.

**Variable**	**Crude Model OR (95%CI) *P*-value**	**Model I OR (95%CI) *P*-value**	**Model II† OR (95%CI) *P*-value**	**Model II ‡ OR (95%CI) *P*-value**
Coffee consumption, cup/d	0.925 (0.916, 0.935) < 0.00001	0.996 (0.985, 1.007) 0.47110	0.999 (0.983, 1.014) 0.86697	0.988 (0.976,1.000) 0.047583
**Coffee consumption categorical, cups/d**				
No intake	Reference	Reference	Reference	Reference
>0, < 1	0.730 (0.679, 0.786) < 0.00001	0.911 (0.834, 0.995) 0.03897	0.851 (0.772, 0.939) 0.00128	0.851 (0.776,0.934) 0.00067
≥1, < 2	0.589 (0.555, 0.624) < 0.00001	0.894 (0.834, 0.959) 0.00172	0.843 (0.780, 0.912) 0.00002	0.829 (0.770,0.892) < 0.00001
≥2, < 3	0.576 (0.537, 0.617) < 0.00001	0.941 (0.868, 1.021) 0.14475	0.896 (0.819, 0.980) 0.01589	0.857 (0.787,0.934) 0.00043
≥3, < 4	0.597 (0.545, 0.655) < 0.00001	0.996 (0.896, 1.107) 0.93732	0.958 (0.854, 1.075) 0.46421	0.920 (0.823,1.028) 0.14040
≥4	0.602 (0.557, 0.650) < 0.00001	0.968 (0.885, 1.059) 0.48259	0.966 (0.874, 1.067) 0.49298	0.923 (0.838,1.016) 0.10128
*P* for trend	< 0.001	0.153	0.175	< 0.001

CI, confidence interval; OR, odds ratio. Models: Crude Model: No covariates adjusted. Model I: Adjusted for sex, age, and race/ethnicity. Model II†: Fully adjusted model with missing data. Model II‡: Fully adjusted model using multiple imputation for missing data. Covariates in fully adjusted models (Model II† and ‡): sex, age, race/ethnicity, poverty-to-income ratio, energy intake, calcium, phosphorus, magnesium, potassium, sodium, education level, body mass index, diabetes, physical activity, smoking status, alcohol consumption, trouble sleeping, chronic kidney disease, supplement taken, and hyperlipidemia.

Stratified analysis (Table 3) identified age as a significant effect modifier of the connection between coffee consumption and hypertension risk (P for interaction < 0.0001). Among individuals aged < 60 years, higher coffee intake was linked to lower odds of hypertension (OR 0.957, 95% CI 0.940–0.975). However, this inverse relationship was not detected in those aged ≥60 years. While sex alone did not show a significant interaction effect, additional stratified analysis by both age and sex revealed that the inverse association remained significant in both males and females under 60, but not in older individuals (Supplementary Table S2). These findings suggest potential age-dependent effects that may differ slightly by sex. No significant interactions were detected for diabetes status, smoking history, or alcohol consumption.

**Table 3:** Relationship between coffee consumption and hypertension subgroups in each.

**Characteristic**	**OR (95%CI)**	***P*-value**	***P* for interaction**
**Age**			< 0.0001
< 60	0.957 (0.940, 0.975)	< 0.0001	
≥ 60	1.023 (0.998, 1.048)	0.0718	
**Sex**			0.1857
Male	1.007 (0.987, 1.026)	0.5028	
Female	0.986 (0.963, 1.011)	0.2769	
**Diabetes**			0.4829
No	0.998 (0.981, 1.015)	0.8059	
Yes Missing	1.008 (0.974, 1.043) 0.757 (0.473, 1.213)	0.6462 0.2471	
**Smoke**			0.2122
Never	0.978 (0.951, 1.006)	0.1275	
Past	0.997 (0.970, 1.024)	0.8168	
Current Missing	1.016 (0.992, 1.040) 1.229 (0.496, 3.046)	0.1920 0.6558	
**Alcohol consumption**			0.8529
Never	0.970 (0.899, 1.047)	0.4334	
Current Past	1.000(0.982,1.018) 1.000 (0.971, 1.031)	0.9822 0.9933	
Missing	0.990 (0.952, 1.028)	0.5921	

Subgroup. CI, confidence interval. OR, odds ratio. Adjusted for sex, age, race/ethnicity, poverty-to-income ratio, energy intake, calcium, phosphorus, magnesium, potassium, sodium, education level, body mass index, diabetes, physical activity, smoking status, alcohol consumption, trouble sleeping, chronic kidney disease, supplement taken, and hyperlipidemia except the subgroup variable.

**Figure 2:**
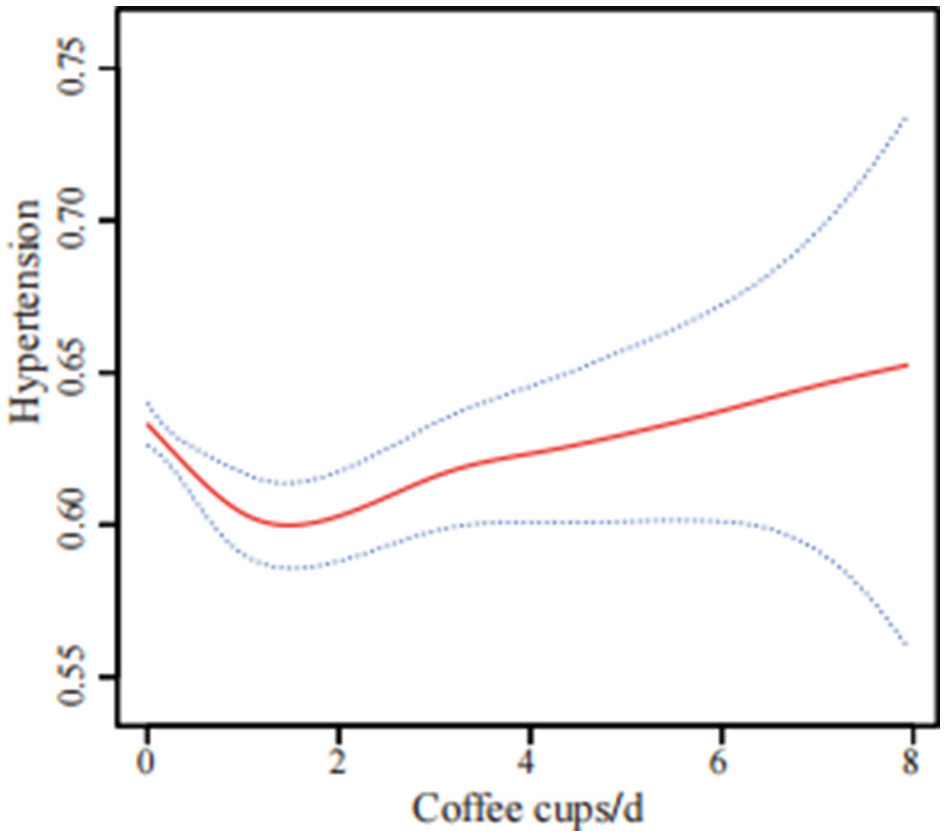
U-shaped association between coffee consumption and hypertension risk using incomplete data (*n* = 22,104). The solid red line shows the smooth curve fitted by a generalized additive model (GAM), and the blue shaded area indicates the 95% confidence interval. The analysis was based on participants with complete data only. The model was adjusted for sex, age, race/ethnicity, poverty to income ratio, energy intake, calcium, phosphorus, magnesium, potassium, sodium, education level, body mass index, diabetes, physical activity, smoking status, alcohol consumption, trouble sleeping, chronic kidney disease, supplement use, and hyperlipidemia.

**Figure 3:**
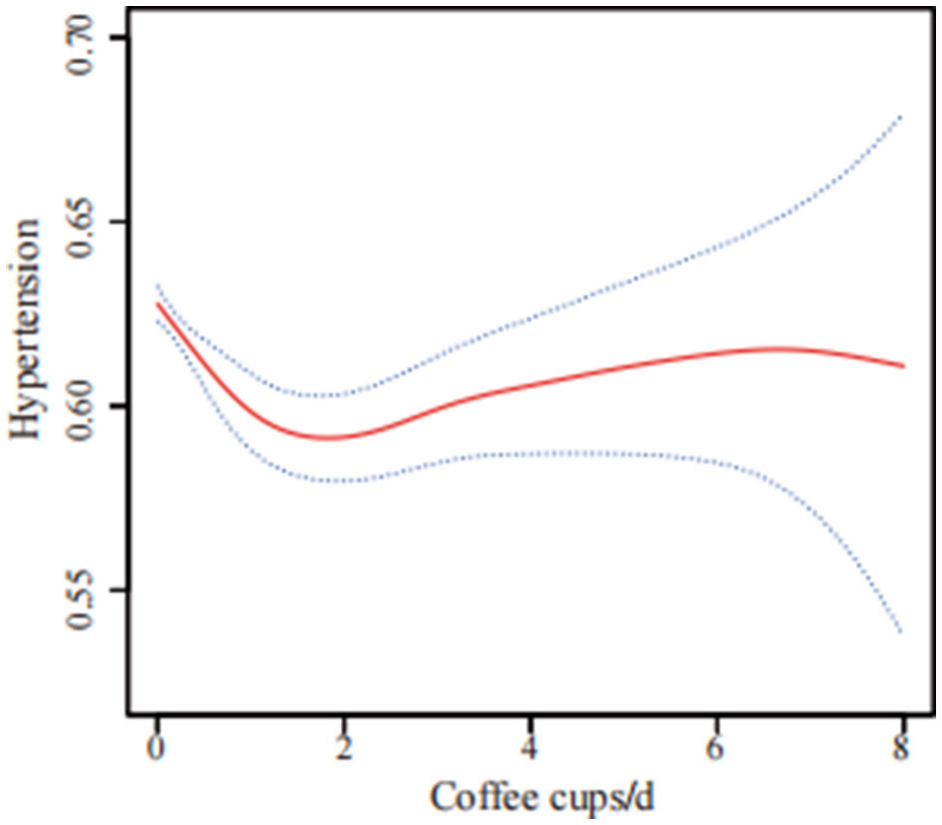
U-shaped association between coffee consumption and hypertension risk using multiple imputation data (*n* = 41,685). The solid red line shows the smooth curve fitted by a generalized additive model (GAM), and the blue shaded area indicates the 95% confidence interval. The analysis was based on data after multiple imputation to account for missing values. The model was adjusted for sex, age, race/ethnicity, poverty to income ratio, energy intake, calcium, phosphorus, magnesium, potassium, sodium, education level, body mass index, diabetes, physical activity, smoking status, alcohol consumption, trouble sleeping, chronic kidney disease, supplement use, and hyperlipidemia.

Curve fitting methods revealed a U-shaped correlation between coffee intake and hypertension risk, with incomplete data (Figure 2) and imputed data (Figure 3). Threshold effect analysis identified an inflection point at 1.71 cups/day in incomplete data (Table 4) and 2.22 cups/day in imputed data (Table 5), indicating a non-linear correlation between coffee intake and the likelihood of developing hypertension. Despite slight variations in thresholds, both analyses confirmed a threshold effect, supporting a non-linear association.

**Table 4:** Threshold effect analysis of coffee consumption (cups/d) and hypertension using incomplete data (*n* = 22,104).

**Models**	**OR (95%CI)**	***P*-value**
**Model I**		
One line effect	0.999 (0.984,1.015)	0.9061
**Model II**		
Inflection point (K)	1.71	
Coffee consumption < 1.7(cups/d)	0.926 (0.883,0.973)	0.0022
Coffee consumption > 1.7(cups/d)	1.022 (1.001,1.044)	0.0396
*P* for log-likelihood ratio test		0.001

Model I, one-line linear regression; Model II, two-segment regression. OR, Odds Ratio; CI, Confidence Interval. Adjusted for sex, age, race/ethnicity, poverty-to-income ratio, energy intake, calcium, phosphorus, magnesium, potassium, sodium, education level, body mass index, diabetes, physical activity, smoking status, alcohol consumption, trouble sleeping, chronic kidney disease, supplement taken, and hyperlipidemia.

**Table 5:** Threshold effect analysis of coffee consumption (cups/d) and hypertension using multiple imputation data (*n* = 41,685).

**Models**	**OR (95%CI)**	***P*-value**
**Model I**		
One line effect	0.988 (0.976,1.000)	0.0503
**Model II**		
Inflection point (K)	2.22	
Coffee consumption < 2.22(cups/d)	0.936 (0.908,0.965)	< 0.0001
Coffee consumption > 2.22(cups/d)	1.012 (0.995,1.030)	0.1792
*P* for log-likelihood ratio test		< 0.001

Model I, one-line linear regression; Model II, two-segment regression. OR, Odds Ratio; CI, Confidence Interval. Adjusted for sex, age, race/ethnicity, poverty-to-income ratio, energy intake, calcium, phosphorus, magnesium, potassium, sodium, education level, body mass index, diabetes, physical activity, smoking status, alcohol consumption, trouble sleeping, chronic kidney disease, supplement taken, and hyperlipidemia.

## Discussion

This large cross-sectional study, utilizing data from eight cycles of the NHANES from 2005 to 2020, investigated the relationship between coffee intake and hypertension among 41,685 participants in the United States. The findings suggest a potential U-shaped relationship, wherein moderate daily coffee intake of up to 2 cups, but not exceeding 3 cups, was linked to a significantly reduced incidence of hypertension contrasted with non-consumers, Notably, age emerged as a significant impact modifier, with the negative link between coffee intake and the risk of hypertension observed only in individuals aged < 60 years. Collectively, these findings suggest that moderate use of coffee might be beneficial for hypertension prevention, particularly in younger adults.

Our findings align with several prior studies that have investigated the link between coffee consumption and hypertension. Xie et al. (22), conducted a meta-analysis that aggregated cohort data, suggesting a negative, dose-dependent relationship between coffee intake and hypertension risk. These findings are further supported by Haghighatdoost et al. (31), who documented an inverse relationship between coffee intake and the risk of hypertension in both cross-sectional and cohort research. Similarly, the SUN Project analysis by Navarro et al. (32), discovered a negative correlation between regular coffee intake and the incidence of hypertension in women. A review by Surma and Oparil (33) concluded that moderate coffee consumption (1–3 cups/day) does not increase and may reduce the possibility of developing hypertension. A study of the ELSA-Brasil cohort by Miranda et al. (34), showed that consuming 1–3 cups/day was related to a reduced risk of hypertension incidence among never smokers.

However, it is crucial to acknowledge that several studies have reported conflicting findings. For instance, a cross-sectional research by Stutz et al. (35) discovered a positive correlation between coffee consumption and an elevated risk of hypertension in individuals with type 1 diabetes. Similarly, Zhang et al. (36) reported in their systematic review that light-to-moderate habitual coffee consumption of 1 to 3 cups per day appeared to be related with a modestly increased risk of hypertension. Additionally, a meta-analysis by Han et al. (37), found no discernible link between coffee consumption and the hypertension risk. Furthermore, Hu et al. (38) discover that there is no significant link between coffee consumption and the risk of hypertension in Finland.

The differences in findings across studies may partly reflect variations in global coffee consumption patterns, such as average intake levels, types of coffee consumed, and methods of preparation. For example, populations in Nordic countries (e.g., Finland) tend to consume large quantities of filtered coffee, while Southern European countries often consume espresso in smaller volumes. In East Asian countries, including China and Japan, coffee consumption has historically been low but is rapidly increasing, often accompanied by different dietary and lifestyle patterns. These regional differences may influence the physiological response to coffee and account for the heterogeneity in observed associations with hypertension risk.

The potential biological mechanisms underlying the relationship between coffee intake and reduced risk of hypertension may be attributed to several key coffee compounds and their effects on established blood pressure regulatory pathways. Chlorogenic acids (CGA), a major group of coffee polyphenols, have been shown to improve endothelial function, enhance nitric oxide production, and inhibit angiotensin-converting enzyme (ACE), thereby promoting vasodilation and reducing vascular resistance (39–41). CGA also exerts antioxidant and anti-inflammatory effects, which may further protect vascular health (39). Other bioactive components in coffee, such as vitamins and essential minerals, may contribute to blood pressure regulation by modulating oxidative stress and systemic inflammation (42–44). Additionally, the interaction between caffeine and CGA may influence insulin sensitivity and sodium balance, both of which are important for blood pressure control (45–47).

The study possesses several noteworthy strengths. Firstly, by utilizing data from eight continuous cycles of the NHANES from 2005 to 2020, the study achieved a large sample size of 41,685 participants, enhancing statistical power and generalizability of the findings. Secondly, the comprehensive data collection, encompassing demographics, socioeconomic status, lifestyle factors, dietary intake, health conditions, and laboratory measurements, enabled the consideration of potential confounding factors. Furthermore, the study employed robust statistical analyses, including multivariable logistic regression models, stratified analyses, and smooth curve fitting techniques, to investigate potential non-linear associations between coffee consumption and hypertension, while adjusting for relevant covariates. Notably, the study analyzed both incomplete and multiple imputed datasets, yielding largely consistent results, thereby enhancing the credibility of the findings and mitigating potential biases arising from missing data.

Nevertheless, the study also presents certain limitations. Firstly, the cross-sectional design precludes the demonstration of causal associations between coffee consumption and hypertension, necessitating longitudinal or interventional studies to determine causality. Secondly, coffee consumption data obtained from 24-h dietary recalls may be prone to recall bias and may not correctly reflect long-term dietary trends. Thirdly, the dataset did not differentiate between caffeinated and decaffeinated coffee, which may have different physiological effects on blood pressure; thus, we were unable to assess their individual associations with hypertension risk. Moreover, despite adjusting for numerous potential confounders, residual confounding from unmeasured or imperfectly measured variables cannot be ruled out. Finally, the study sample was limited to the people in the United States, and the generalizability of the findings may be influenced by different dietary habits, environmental exposures, and genetic backgrounds in other populations.

## Conclusions

In conclusion, our cross-sectional study indicates that moderate coffee consumption specifically in an average of 1–3 cups per day, may be linked to a lower prevalence of hypertension compared to non-drinkers. Interestingly, this association was found to be modified by age, with the protective effect of coffee consumption being more pronounced in individuals younger than 60 years old. However, due to the cross-sectional nature of the analysis, no causal inferences can be drawn. Future prospective cohort research and randomized controlled studies are warranted to confirm these associations and to determine the optimal amount and type of coffee consumption for potential hypertension prevention.

## Data Availability

Publicly available datasets were analyzed in this study. This data can be found here: the original data can be obtained from NHANES (https://www.cdc.gov/nchs/nhanes/about/index.html).
