# Supplementary material for: A cross-sectional analysis of coffee intake and hypertension prevalence: results from the NHANES 2005–2020
Source: Front Nutr. 2025 Aug 11;12:1615528. doi: 10.3389/fnut.2025.1615528 (PMC12375616; doi:10.3389/fnut.2025.1615528)
Supplement: Supplementary file 1 [file Table_1.docx]

Table S1. Univariate Analysis for hypertension

| Covariate | Statistics | Hypertension  OR (95% CI) | P-value |
| --- | --- | --- | --- |
| Coffee consumption, cups/d | 1.076 ± 2.033 | 0.925 (0.916, 0.935) | <0.00001 |
| BMI, (kg/m2) | 28.832 ± 7.385 | 0.928 (0.925, 0.931) | <0.00001 |
| Energy, kcal/d | 2103.278 ± 989.441 | 1.000 (1.000, 1.000) | <0.00001 |
| Calcium, mg/d | 923.687 ± 582.787 | 1.000 (1.000, 1.000) | <0.00001 |
| Phosphorus, mg/d | 1330.384 ± 673.452 | 1.000 (1.000, 1.000) | <0.00001 |
| Magnesium, mg/d | 289.350 ± 148.019 | 1.001 (1.001, 1.001) | <0.00001 |
| Potassium, mg/d | 2563.514 ± 1257.641 | 1.000 (1.000, 1.000) | <0.00001 |
| Sodium, mg/d | 3421.605 ± 1810.640 | 1.000 (1.000, 1.000) | <0.00001 |
| Age, years | 46.467 ± 20.057 | 0.935 (0.934, 0.937) | <0.00001 |
| PIR, % | 2.504 ± 1.626 | 1.000 (0.987, 1.013) | 0.99767 |
| Sex, % |  |  |  |
| Female | 21754 (52.187%) | Reference |  |
| Male | 19931 (47.813%) | 0.939 (0.902, 0.977) | 0.00169 |
| Race and ethnicity, % |  |  |  |
| Non-Hispanic Black | 9655 (23.162%) | Reference |  |
| Non-Hispanic White | 17167 (41.183%) | 1.278 (1.216, 1.344) | <0.00001 |
| Other Hispanic | 3916 (9.394%) | 1.706 (1.579, 1.843) | <0.00001 |
| Mexican American | 6404 (15.363%) | 2.308 (2.157, 2.470) | <0.00001 |
| Other Race - Including Multi-Racial | 4543 (10.898%) | 2.132 (1.978, 2.299) | <0.00001 |
| Education, % |  |  |  |
| Less than high school | 5443 (13.057%) | Reference |  |
| High school or equivalent | 14800 (35.504%) | 0.589 (0.552, 0.630) | <0.00001 |
| College or above | 21082 (50.575%) | 0.719 (0.674, 0.766) | <0.00001 |
| Missing | 360 (0.864%) | 4.215 (2.947, 6.028) | <0.00001 |
| Smoking, % |  |  |  |
| Never | 21843 (52.400%) | Reference |  |
| Past | 9333 (22.389%) | 0.507 (0.482, 0.532) | <0.00001 |
| Current | 7335 (17.596%) | 0.883 (0.837, 0.933) | <0.00001 |
| Missing | 3174 (7.614%) | 19.691 (15.957, 24.300) | <0.00001 |
| Trouble sleeping, % |  |  |  |
| No | 29338 (70.380%) | Reference |  |
| Yes | 10353 (24.836%) | 0.469 (0.448, 0.490) | <0.00001 |
| Missing | 1994 (4.783%) | 121.409 (63.102, 233.593) | <0.00001 |
| Diabetes, % |  |  |  |
| No | 33830 (81.156%) | Reference |  |
| Yes | 7185 (17.236%) | 0.188 (0.178, 0.199) | <0.00001 |
| Missing | 670 (1.607%) | 5.161 (3.922, 6.790) | <0.00001 |
| Alcohol drinking, % |  |  |  |
| Never | 5042 (12.095%) | Reference |  |
| Current | 24894 (59.719%) | 1.164 (1.094, 1.238) | <0.00001 |
| Past | 4850 (11.635%) | 0.555 (0.512, 0.601) | <0.00001 |
| Missing | 6899 (16.550%) | 1.860 (1.722, 2.010) | <0.00001 |
| Activity, % |  |  |  |
| Inactive | 6726 (16.135%) | Reference |  |
| Active | 22837 (54.785%) | 1.332 (1.260, 1.408) | <0.00001 |
| Missing | 12122 (29.080%) | 0.921 (0.867, 0.978) | 0.00760 |
| CKD |  |  |  |
| No | 30658 (73.547%) | Reference |  |
| Yes | 7186 (17.239%) | 0.249 (0.236, 0.263) | <0.00001 |
| Missing | 3841 (9.214%) | 1.861 (1.717, 2.017) | <0.00001 |
| Hyperlipidemia |  |  |  |
| No | 14299 (34.303%) | Reference |  |
| Yes | 27382 (65.688%) | 0.339 (0.324, 0.355) | <0.00001 |
| Missing | 4 (0.010%) | 0.099 (0.010, 0.947) | 0.04478 |
| Coffee consumption categorical, cups/d |  |  |  |
| No intake | 23866 (57.253%) | Reference |  |
| >0, <1 | 3391 (8.135%) | 0.730 (0.679, 0.786) | <0.00001 |
| ≥1, <2 | 5740 (13.770%) | 0.589 (0.555, 0.624) | <0.00001 |
| ≥2, <3 | 3786 (9.082%) | 0.576 (0.537, 0.617) | <0.00001 |
| ≥3, <4 | 2010 (4.822%) | 0.597 (0.545, 0.655) | <0.00001 |
| ≥4 | 2892 (6.938%) | 0.602 (0.557, 0.650) | <0.00001 |
| Supplement taken |  |  |  |
| No | 20644 (49.542%) | Reference |  |
| Yes | 21026 (50.458%) | 0.570 (0.547, 0.593) | <0.00001 |

## Abbreviations: CI, confidence interval. OR, odds ratio. BMI, body mass index; CKD, chronic kidney disease. PIR, poverty to income ratio
