# Supplementary material for: A cross-sectional analysis of coffee intake and hypertension prevalence: results from the NHANES 2005–2020
Source: Front Nutr. 2025 Aug 11;12:1615528. doi: 10.3389/fnut.2025.1615528 (PMC12375616; doi:10.3389/fnut.2025.1615528)
Supplement: Supplementary file 2 [file Table_2.docx]

Table S2. Association between coffee consumption and hypertension risk, stratified by sex and age.

| Sex | Age Group | OR (95%CI) | *P-*value | *P* for interaction |
| --- | --- | --- | --- | --- |
| Female | <60 years | 0.945 (0.915–0.976) | 0.0005 | 0.0193 |
|  | ≥60 years | 1.004 (0.965–1.044) | 0.8527 |  |
| Male | <60 years | 0.966 (0.945–0.988) | 0.0022 | 0.0008 |
|  | ≥60 years | 1.034 (0.999–1.070) | 0.0562 |  |

Abbreviations: CI, confidence interval. OR, odds ratio.

Adjusted for race/ethnicity, poverty-to-income ratio, energy intake, calcium, phosphorus, magnesium, potassium, sodium, education level, body mass index, diabetes, physical activity, smoking status, alcohol consumption, trouble sleeping, chronic kidney disease, supplement taken, and hyperlipidemia.
